# Characteristics of the corpus callosum in chronic schizophrenia treated with clozapine or risperidone and those never-treated

**DOI:** 10.1186/s12888-021-03552-0

**Published:** 2021-10-30

**Authors:** Bo Tao, Yuan Xiao, Hengyi Cao, Wenjing Zhang, Chengmin Yang, Rebekka Lencer, Qiyong Gong, Su Lui

**Affiliations:** 1 Huaxi MR Research Center (HMRRC), Functional and Molecular Imaging Key Laboratory of Sichuan Province, Department of Radiology, West China Hospital, Sichuan University, 37 Guo Xuexiang, Chengdu, 610041 China; 2 Psychoradiology Research Unit of the Chinese Academy of Medical Sciences, West China Hospital of Sichuan University, Chengdu, China; 3 Center for Psychiatric Neuroscience, Feinstein Institute for Medical Research, Manhasset, NY USA; 4 Division of Psychiatry Research, Zucker Hillside Hospital, Glen Oaks, NY USA; 5 Department of Psychiatry and Psychotherapy, University of Lübeck, Lübeck, Germany

**Keywords:** corpus callosum (CC), Magnetic resonance imaging (MRI), Diffusion tensor imaging (DTI), Fractional anisotropy (FA), Clozapine, Risperidone

## Abstract

**Background:**

The corpus callosum (CC) deficits have been well documented in chronic schizophrenia. However, the long-term impacts of antipsychotic monotherapies on callosal anatomy remain unclear. This cross-sectional study sought to explore micro- and macro-structural characteristics of the CC in never-treated patients and those with long-term mono-antipsychotic treatment.

**Methods:**

The study included 23 clozapine-treated schizophrenia patients (CT-SCZ), 19 risperidone-treated schizophrenia patients (RT-SCZ), 23 never-treated schizophrenia patients (NT-SCZ), and 35 healthy controls (HCs). High resolution structural images and diffusion tensor imaging (DTI) data for each participant were obtained via a 3.0 T MR scanner. FreeSurfer was used to examine the volumes and fractional anisotropy (FA) values of the CC for each participant.

**Results:**

There were significant deficits in the total and sub-regional CC volume and white matter integrity in NT-SCZ in comparison with healthy subjects. Compared with NT-SCZ, both CT-SCZ and RT-SCZ showed significantly increased FA values in the anterior CC region, while only RT-SCZ showed significantly increased volume in the mid-anterior CC region. Moreover, the volume of the mid-anterior CC region was significantly smaller in CT-SCZ compared to HCs. No correlations of clinical symptoms with callosal metrics were observed in schizophrenia patients.

**Conclusions:**

Our findings provide insight into micro- and macro-structural characteristics of the CC in chronic schizophrenia patients with or without antipsychotics. These results suggest that the pathology itself is responsible for cerebral abnormalities in schizophrenia and that chronic exposure to antipsychotics may have an impact on white matter structure of schizophrenia patients, especially in those with risperidone treatment.

## Background

The corpus callosum (CC) is the largest commissural fiber in human brain and implicated in the etiopathology of schizophrenia [1–4]. However, little is known regarding the effects of antipsychotic medications on callosal anatomy in schizophrenia patients over long-term treatment [5, 6]. Compared with healthy controls (HCs), a recent study found increased volume of the posterior region of the CC in patients with long-term exposure to antipsychotic medications [5]. Moreover, patients with poor outcome after treatment had more pronounced reduction in CC size compared to good-outcome patients and HCs [6]. Notably, neither of these studies included long-term drug-naive patients as a comparison group, complicating the interpretation of the findings as whether they reflect effects of neuropathology or medication remains unclear.

Prior studies have demonstrated that the choice of antipsychotics may display different impacts on white matter structure in schizophrenia in terms of their different pharmacological mechanisms [7, 8]. Clozapine and risperidone, both of which are atypical antipsychotic medications, have been widely prescribed in clinical practice, but their pharmacological mechanisms are not exactly the same. Clozapine is the only antipsychotic medication approved for treatment-resistant schizophrenia in most countries [9]. It has a high affinity to dopamine D4 receptors and other receptors such as 5­HT2A, 5­HT1C, adrenergic receptors [10]. Risperidone is another atypical antipsychotic that is antagonistic to 5­HT2 and dopamine D2 receptors [10]. In contrast, clozapine has a low affinity to dopamine D2 receptors [10]. In addition, our previous study on chronic schizophrenia showed less alterations in white matter structural networks in risperidone-treated patients compared with clozapine-treated or never-treated patients [11]. However, few studies have explored the effects of long-term monotherapy with clozapine or risperidone on callosal structure in chronic schizophrenia.

In the present study, we combined structural magnetic resonance imaging (MRI) and diffusion tensor imaging (DTI) to examine the impacts of long-term clozapine and risperidone therapies on the micro- and macrostructures [fractional anisotropy (FA) & volume respectively] of the CC. According to our previous findings [11, 12], we hypothesized that antipsychotic-treated schizophrenia patients would show larger volumes and/or increased FA values of the CC compared with never-treated patients, especially in those with long-term risperidone monotherapy.

## Methods

### Participants

The study was a cross-sectional design and recruited forty-two chronic schizophrenia patients with long-term mono-antipsychotic treatments, including 23 clozapine-treated schizophrenia patients (CT-SCZ) and 19 risperidone-treated schizophrenia patients (RT-SCZ), 23 never-treated chronic schizophrenia patients, and 35 HCs. Clinical diagnoses of schizophrenia were made by an experienced psychiatrist based on the Structured Clinical Interview for DSM-IV Axis I Disorders (SCID). The Nottingham Onset Schedule was conducted to evaluate illness onset and duration using information provided by patients, family members and available medical records [13]. The Positive and Negative Symptom Scale (PANSS) was used to estimate the severity of clinical symptoms of the patients [14].

Antipsychotic-treated patients were recruited from the local psychiatric clinic. All participants had received either clozapine or risperidone monotherapy consistently for over five years before entry into the study based on intact medical records. Never-treated schizophrenia patients (NT-SCZ) were recruited from a mental health screening program designed to provide psychiatric care to individuals with serious but untreated mental illness in rural areas around the Chengdu City of China. They did not receive any antipsychotic medications due to many factors, including family stigma, the lack of understanding of the severity of mental illness, and poor socioeconomic conditions.

HCs were recruited from the same community via poster advertisements. The non-patient edition of the SCID was used to ensure lifetime absence of psychotic, anxiety and mood disorders. Individuals with a known family history of major psychiatric illness in their first or second-degree relatives were excluded. All participants were right-handed and met the following inclusion criteria: 1) no history of substance abuse or dependence, 2) no history of significant systemic illness, head injury or neurologic illness, and 3) no contraindication to MR scanning.

The study methods were performed in accordance with the relevant guidelines and regulations. Ethical approval for this study was approved by the research ethics committee of West China Hospital of Sichuan University. All participants provided written informed consent for study procedures, and informed consent for schizophrenia patients was obtained from their parents or legal guardians.

### MR image acquisition

MRI examination of each subject was performed via a 3.0 T GE Signa EXCITE scanner (General Electric, Miwaukee, Wisconsin) with 8-channel phase array head coil. DTI images were obtained using a bipolar diffusion single-shot echo planar imaging sequence. Each DTI dataset included 15 images of non-collinear directions (b = 1000 s/mm^2^) with a reference image without diffusion weighting (b = 0). The acquisition parameters were as follows: TR = 10,000 ms, TE = 70 ms, flip angle = 90°, field of view = 24 × 24 cm^2^, matrix size = 128 × 128, number of axial slice = 50; and slice thickness = 3 mm (no gap). High resolution T1-weighted images for registration were obtained using a three-dimensional spoiled gradient (SPGR) sequence. The acquisition parameters were as follows: TR = 8.5 ms, TE = 3.4 ms, TI = 400 ms, flip angle = 12°, field of view = 24 × 24 cm^2^, matrix size = 256 × 256 × 128, number of axial slice = 156; and slice thickness = 1 mm (no gap).

### Imaging preprocessing and corpus callosum measurements

**Fig. 1:**
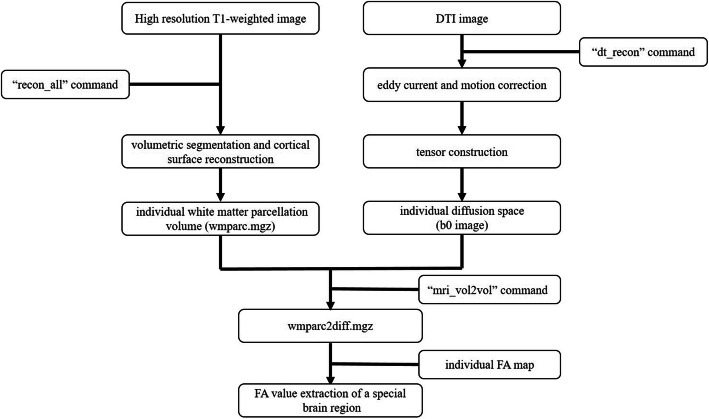
The preprocessing pipeline of imaging data. Note: DTI, diffusion tensor imaging; FA, fractional anisotropy

FreeSurfer was used to preprocess imaging data, and to automatically identify and segment CC for each participant, which could reduce random errors, rater errors and inter-participant variability of manual editing [12]. The preprocessing pipeline of imaging data was displayed carefully in Fig. 1. Firstly, we conducted automated volumetric segmentation and cortical surface reconstruction of each high resolution T1-weighted data using the standard “recon_all” processing stream, as described carefully in other studies [15, 16]. Then, volumetric segmentation and cortical surface reconstruction of each participant were examined carefully in a multiple view to identify whether there were too much or not enough removed regions. Next, the “dt_recon” command was used to preprocess diffusion-weighted images based on individual anatomical data derived from the “recon_all” procedure. It was performed via an orderly process that included eddy current and motion correction employing FSL’s eddy_ correct, tensor construction and intra-subject registrations in native space. This yielded individual FA map, b0 image and other relevant maps. After visually checking the registration, we resampled individual white matter parcellation volume (wmparc.mgz) in its’ diffusion space (b0 image) using the “mri_vol2vol” command, outputting a new segmentation volume named “wmparc2diff.mgz”. Lastly, the “wmparc2diff.mgz” volume of each subject was applied to the corresponding FA map to analyze white matter integrity of a special brain region.

**Fig. 2:**
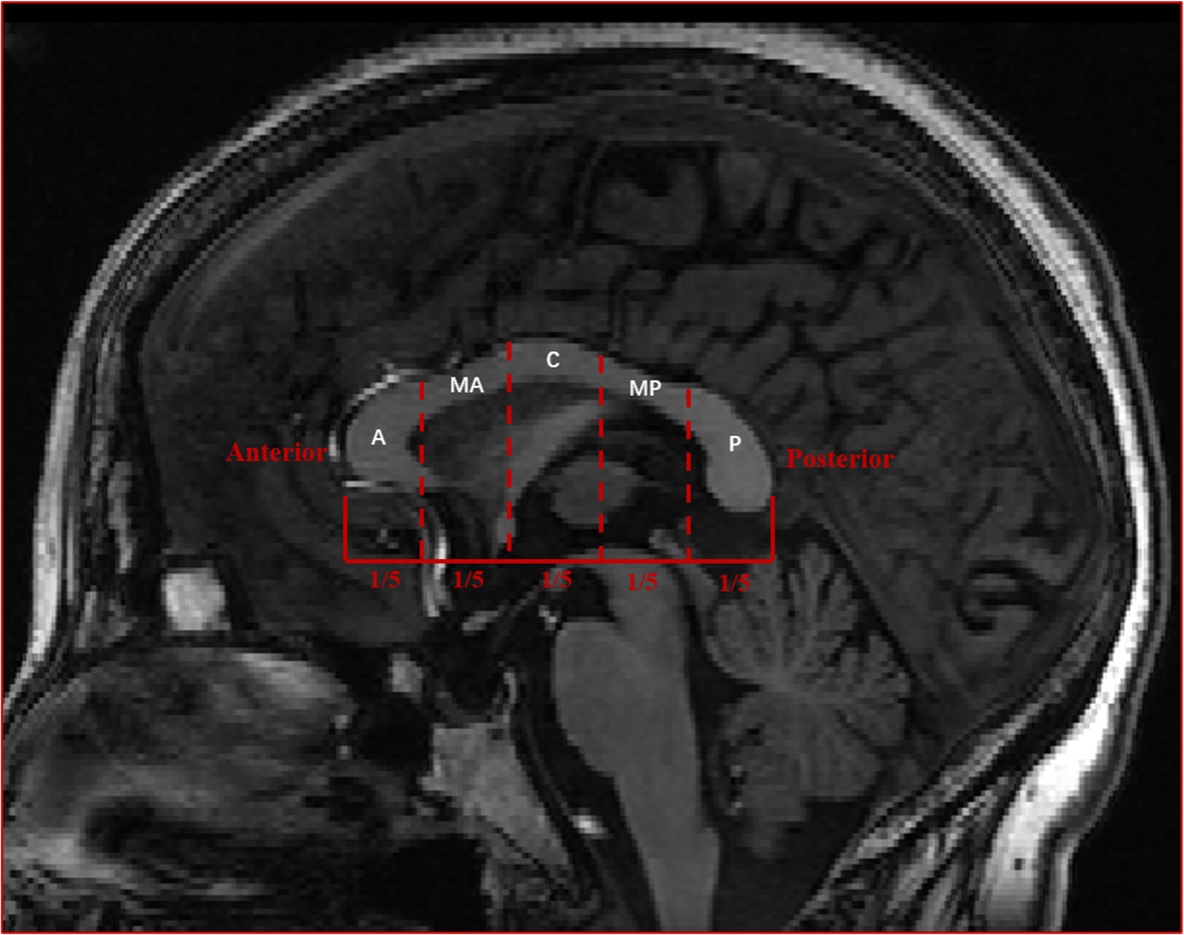
Corpus callosum was divided into five components of equal length along its’ primary eigendirection, corresponding to functional subdivisions, namely: anterior, mid-anterior, central, mid-posterior and posterior portions. Note: A, anterior (rostrum); MA, mid-anterior (genu); C, central (truncus/body); MP, mid-posterior (anterior splenium); P, posterior (posterior splenium)

The CC of each subject was automatically divided into five components of equal length along the primary eigen direction using FreeSurfer, corresponding to its functional subdivisions, namely: anterior, mid-anterior, central, mid-posterior and posterior portions (Fig. 2) [17]. Total and subregional volumes of the CC were extracted via FreeSurfer. It was related with the lateral extent where white matter tracts that run horizontally from the mid-sagittal plane of the CC change direction bilaterally [17]. Moreover, FA values of five CC subregions were extracted according to the boundary defined by the CC volume segmentation.

### Total intracranial volume (TIV)

We extracted total intracranial volume (TIV) (mm^3^) of each participant in individual native space using FreeSurfer [18].

### Statistical analysis

One-way analysis of variance (ANOVA), two sample t-tests or chi-square tests were used to compare the demographic and clinical characteristics of the sample, including age, sex, education, illness duration, medications, TIV and PANSS scores.

Before analyzing anatomical characteristics of the CC, we firstly removed extreme outliers in the study (individual volume or FA values greater than/less than 3 standard deviations from group mean). Then, we test the interactions of diagnostic group with sex or age for callosal volumes and FA values using general linear model (GLM). Multivariate analysis of covariance (MANCOVA) was used to test for between-group differences in subregional volumes and FA values of the CC. Univariate analysis of covariance (ANCOVA) was used to test for group differences in CC total volume. In these statistical models, diagnostic group (CT-SCZ, RT-SCZ, NC-SCHZ, HCs) and sex (females vs. males) were considered as between-subject factors, and age, years of education and daily chlorpromazine equivalent dosage were regarded as covariates [17]. Two-sample t tests with false discovery rate (FDR) correction were performed for post-hoc comparisons once significant main effects were observed. We also examined correlations of clinical symptoms with statistically significant group differences in callosal metrics using Pearson correlation analysis. All analyses were performed using SPSS (version 18.0) and statistical significance was set at two-tailed *P* <  0.05.

## Results

### Demographic data

The clinical and demographic data are displayed in Table 1. Age, sex, and TIV were not significantly different among groups, though there was a significant difference in education (*P* = 0.001). PANSS total and subscale scores in NT-SCZ were significantly higher than treated patients (*P* <  0.001) and there was no significant difference in PANSS scores between RT-SCZ and CT-SCZ. In addition, mean daily chlorpromazine equivalent doses in RT-SCZ were significantly larger than that in CT-SCZ (*P* = 0.019).

**Table 1:** Demographic and clinical characteristics of study participants

	CT-SCZ, *N* = 23 Mean ± SD	RT-SCZ, *N* = 19 Mean ± SD	NT-SCZ, *N* = 23 Mean ± SD	HCs, *N* = 35 Mean ± SD	Statistics (ANOVA, t or χ2)	*p* value	Post-hoc analyses					
							NT-SCZ vs. HCs	NT-SCZ vs. RT- SCZ	NT-SCZ vs. CT-SCZ	RT-SCZ vs. CT- SCZ	RT-SCZ vs. HCs	CT-SCZ vs. HCs
Age (years)	48.96 ± 6.76	43.63 ± 7.83	44.26 ± 13.619	44.29 ± 2.44	2.105	0.105	NS	NS	NS	0.038*	NS	0.036*
Gender (M/F)	14/9	11/8	12/11	13/22	3.918	0.270	NS	NS	NS	NS	NS	NS
Education (years)	9.43 ± 2.84	10.58 ± 3.37	6.83 ± 4.78	8.01 ± 1.77	5.634	**0.001**	NS	< 0.001***	0.001**	NS	0.006**	NS
Illness duration (years)	18.34 ± 7.67	14.11 ± 8.43	16.44 ± 10.88	–	1.114	0.345	–	NS	NS	NS	–	–
PANSS score												
Total	57.21 ± 11.42	49.44 ± 12.99	92.26 ± 19.53	–	42.681	**< 0.001**	–	< 0.001***	< 0.001***	NS	–	–
Positive symptoms	10.79 ± 3.09	9.61 ± 2.91	23.09 ± 6.61	–	47.737	**< 0.001**	–	< 0.001***	< 0.001***	NS	–	–
Negative symptoms	18.79 ± 5.59	14.83 ± 5.92	25.57 ± 9.59	–	10.374	**< 0.001**	–	< 0.001***	< 0.001***	NS	–	–
General psychopathological symptoms	27.64 ± 5.32	24.83 ± 5.59	43.61 ± 8.47	–	43.858	**< 0.001**	–	< 0.001***	< 0.001***	NS	–	–
Chlorpromazine equivalents (mg/day)	233.99 ± 101.62	316.25 ± 115.46	–	–	2.455	**0.019**	–	–	–	–	–	–
Total Intracranial Volume (TIV) (cm ^3^)	1431.73 ± 159.18	1442.26 ± 175.39	1401.05 ± 132.88	1394.01 ± 224.44	0.401	0.753	NS	NS	NS	NS	NS	NS

Note: *ANOVA* analysis of variance, *CT-SCZ* clozapine-treated schizophrenia patients, *RT-SCZ* risperidone-treated schizophrenia patients, *NT-SCZ* never-treated schizophrenia patients, *HCs* healthy controls, *PANSS* Positive and Negative Syndrome Scale, *NS* not significant, *, Significant group difference at *P* < 0.05; **, *P* < 0.01; ***, *P* < 0.001

### Groups differences in callosal volumes

**Fig. 3:**
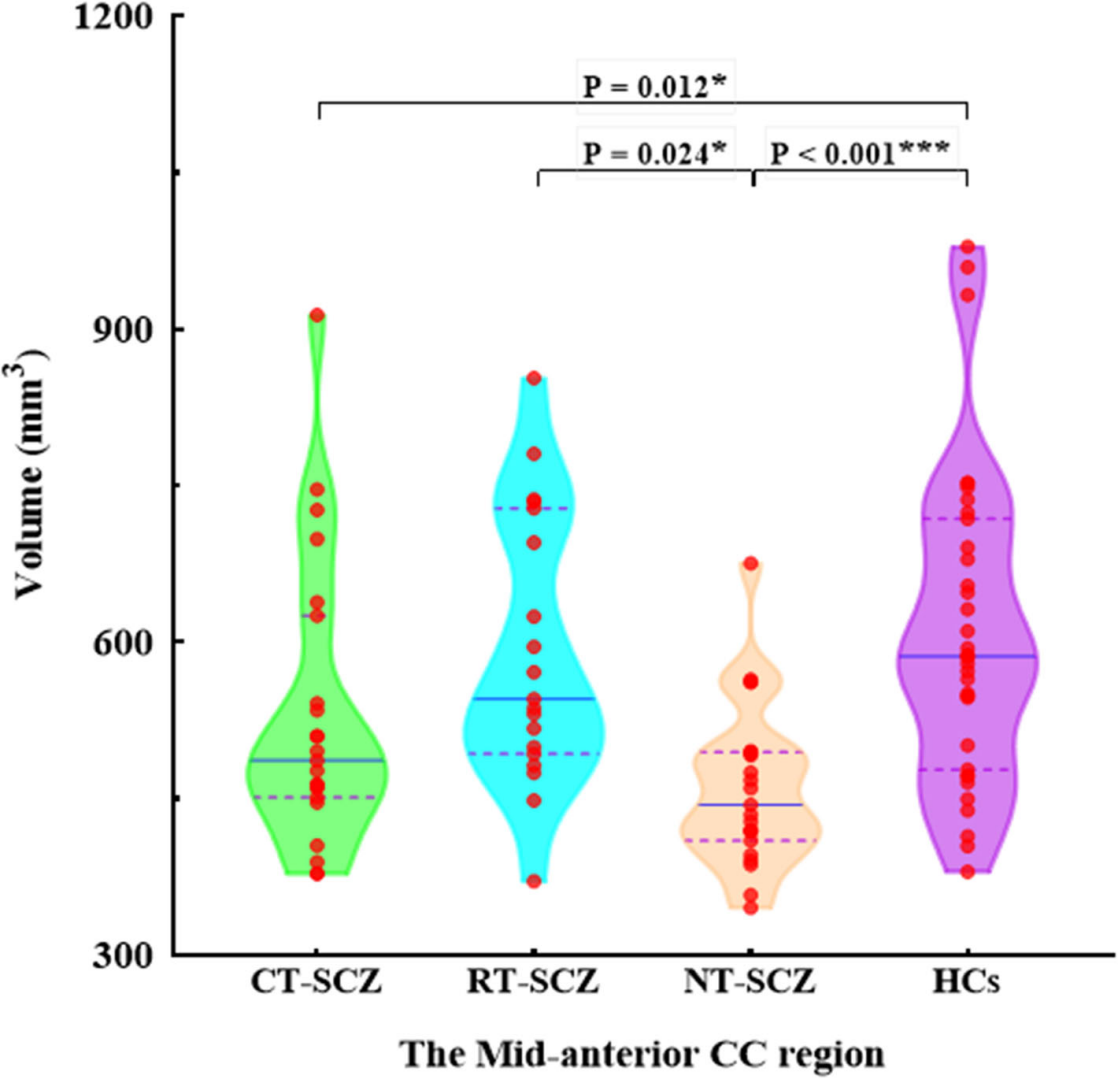
Volume (mm^3^) of the mid-anterior CC region in CT-SCZ, RT-SCZ, NT-SCZ and HCs. Note: CT-SCZ, clozapine-treated schizophrenia patients; RT-SCZ, risperidone-treated schizophrenia patients; NT-SCZ, never-treated schizophrenia patients; HCs, healthy controls; CC, corpus callosum; *, Significant group difference at *P* < 0.05; **, *P* < 0.01; ***, *P* < 0.001

There were no any significant interactions between sex/age and diagnostic group for callosal volumes. Univariate ANCOVA showed a significant main effect of diagnostic group on total CC volume (*F* = 3.213, *P* = 0.027). Pairwise comparison analyses revealed that NT-SCZ had significantly reduced CC volume compared to HCs (*P* = 0.004, *P* <  0.05 corrected with FDR) (Table 2), while the other groups did not significantly differ from each other. MANCOVA of subregional volumes of the CC showed a significant main effect of diagnostic group (*F* = 2.228, *P* = 0.006), where significant differences in the volume of the mid-anterior CC region were shown among the four groups (*F* = 6.380, *P* = 0.001) (Fig. 3). Specifically, post-hoc analyses showed significantly higher mid-anterior CC volume in HCs compared with both NT-SCZ (*P* <  0.001, *P* <  0.05 corrected with FDR) and CT-SCZ (*P* = 0.012, *P* <  0.05 corrected with FDR). Moreover, RT-SCZ had significantly increased volume compared to NT-SCZ (*P* = 0.024, *P* <  0.05 corrected with FDR). MANCOVA test also showed a significant difference in the volume of the central CC region among the four participant groups (*F* = 3.318, *P* = 0.023). Post-hoc analyses revealed that such difference was between HCs and NT-SCZ (*P* = 0.006, *P* <  0.05 corrected with FDR). There were no significant group differences in the volumes of other three CC subregions.

**Table 2:** Corpus callosum volumes (mm^3^) in CT-SCZ, RT-SCZ, NT-SCZ and HCs

Corpus callosum	CT-SCZ, ***N*** = 23 Mean ± SD	RT-SCZ, ***N*** = 19 Mean ± SD	NT-SCZ, ***N*** = 23 Mean ± SD	HCs, ***N*** = 35 Mean ± SD	F value	***p*** value	Post-hoc analyses					
							NT-SCZ vs. HCs	NT-SCZ vs. RT-SCZ	NT-SCZ vs. CT- SCZ	RT-SCZ vs. CT- SCZ	RT-SCZ vs. HCs	CT-SCZ vs. HCs
**Anterior**	916.73 ± 145.59	866.72 ± 155.71	831.76 ± 110.77	845.43 ± 124.69	0.601	0.616	NS	NS	NS	NS	NS	NS
**Mid-anterior**	530.28 ± 135.11	590.23 ± 126.91	460.38 ± 76.92	611.49 ± 150.69	6.380	**0.001**	< 0.001***	0.024*	NS	NS	NS	0.012*
**Central**	546.32 ± 152.74	538.48 ± 123.73	490.27 ± 114.93	600.16 ± 139.44	3.318	**0.023**	0.006**	NS	NS	NS	NS	NS
**Mid-posterior**	479.59 ± 96.39	500.46 ± 107.27	454.47 ± 87.86	503.92 ± 78.22	1.287	0.284	NS	NS	NS	NS	NS	NS
**Posterior**	942.36 ± 157.09	949.00 ± 172.78	835.62 ± 128.31	900.63 ± 148.86	1.995	0.120	NS	NS	NS	NS	NS	NS
**Total**	3415.29 ± 524.04	3444.89 ± 553.07	3072.52 ± 363.73	3450.67 ± 414.81	3.213	**0.027**	0.004**	NS	NS	NS	NS	NS

**Note:** *CT-SCZ* clozapine-treated schizophrenia patients, *RT-SCZ* risperidone-treated schizophrenia patients, *NT-SCZ* never-treated schizophrenia patients, *HCs* healthy controls, *NS* not significant, *, Significant group difference at *P* < 0.05; **, *P* < 0.01; ***, *P* < 0.001

### Groups differences in callosal FA values

**Fig. 4:**
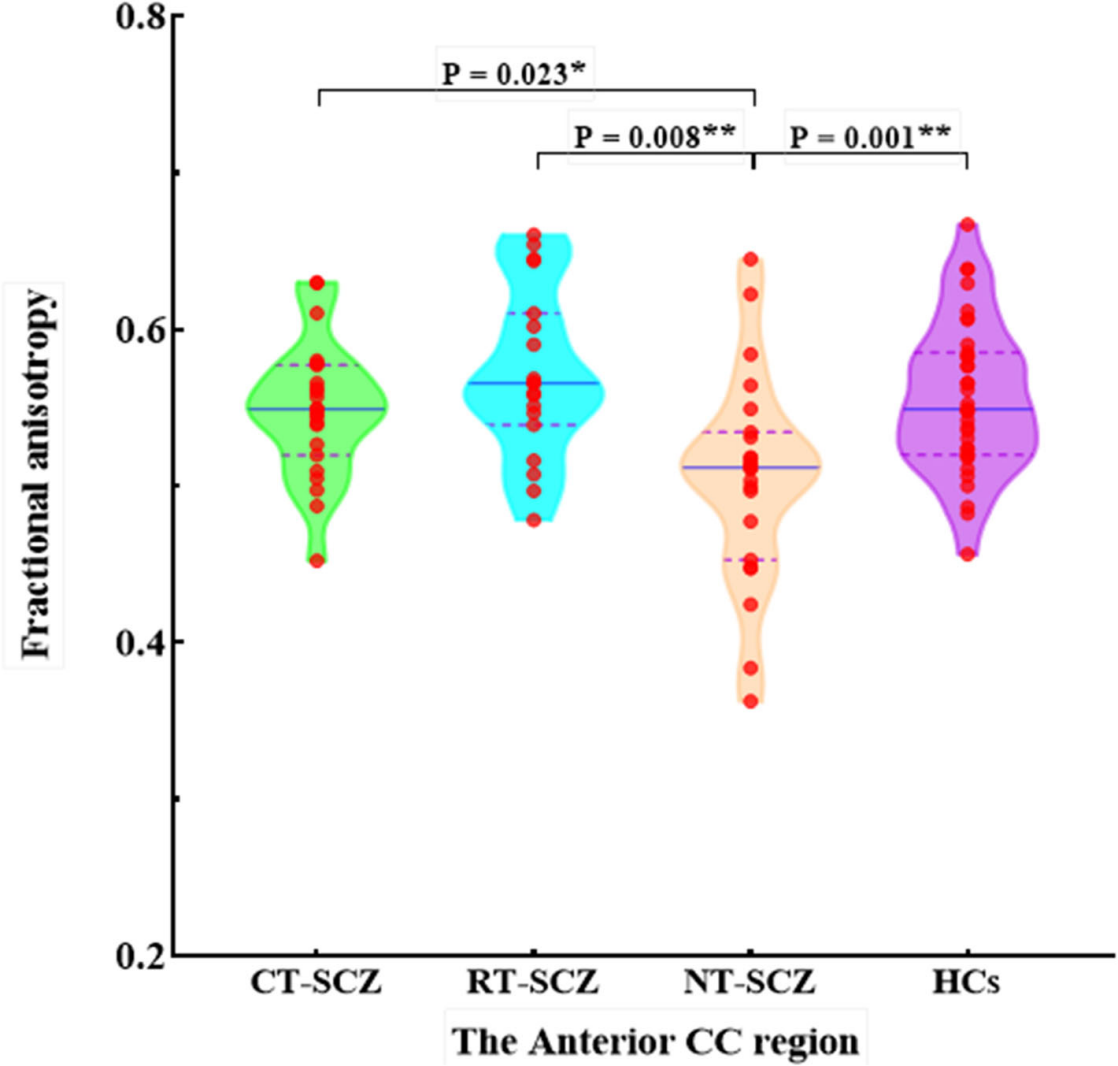
Fractional anisotropy (FA) of the anterior CC region in CT-SCZ, RT-SCZ, NT-SCZ and HCs. Note: CT-SCZ, clozapine-treated schizophrenia patients; RT-SCZ, risperidone-treated schizophrenia patients; NT-SCZ, never-treated schizophrenia patients; HCs, healthy controls; CC, corpus callosum; *, Significant group difference at *P* < 0.05; **, *P* < 0.01; ***, *P* < 0.001

FA values in the five CC subregions are listed in Table 3. No interactions of diagnostic group with sex or age for callosal FA values were statistically different. MANCOVA test showed a significant main effect of diagnostic group (*F* = 1.723, *P* = 0.047), and there were significant differences in FA values of the anterior CC region among the four groups (*F* = 4.761, *P* = 0.004) (Fig. 4). Post-hoc analyses showed that the FA values of the anterior CC region in HCs were significantly greater than that in NT-SCZ (*P* = 0.001, *P* <  0.05 corrected with FDR), while there were no significant differences in FA values of this CC region between treated patients and HCs. In addition, both RT-SCZ and CT-SCZ had significantly increased FA values in the anterior region of the CC in contrast with NT-SCZ (*P* = 0.008 & *P* = 0.023, *P* <  0.05 corrected with FDR). There were no significant group differences in FA values of other four CC subregions.

**Table 3:** Corpus callosum fractional anisotropy (FA) values in CT-SCZ, RT-SCZ, NT-SCZ and HCs

Corpus callosum	CT-SCZ, ***N*** = 23 Mean ± SD	RT-SCZ, ***N*** = 19 Mean ± SD	NT-SCZ, ***N*** = 23 Mean ± SD	HCs, ***N*** = 35 Mean ± SD	F value	***p*** value	Post-hoc analyses					
							NT-SCZ vs. HCs	NT-SCZ vs. RT-SCZ	NT-SCZ vs. CT- SCZ	RT-SCZ vs. CT- SCZ	RT-SCZ vs. HCs	CT-SCZ vs. HCs
**Anterior**	0.54 ± 0.04	0.57 ± 0.05	0.50 ± 0.06	0.55 ± 0.04	4.761	**0.004**	0.001**	0.008**	0.023*	NS	NS	NS
**Mid-anterior**	0.43 ± 0.06	0.42 ± 0.04	0.43 ± 0.05	0.42 ± 0.04	0.530	0.663	NS	NS	NS	NS	NS	NS
**Central**	0.45 ± 0.08	0.45 ± 0.06	0.47 ± 0.05	0.47 ± 0.06	0.342	0.759	NS	NS	NS	NS	NS	NS
**Mid-posterior**	0.41 ± 0.07	0.42 ± 0.07	0.42 ± 0.06	0.41 ± 0.04	1.016	0.389	NS	NS	NS	NS	NS	NS
**Posterior**	0.60 ± 0.05	0.64 ± 0.06	0.63 ± 0.06	0.63 ± 0.04	0.972	0.409	NS	NS	NS	NS	NS	NS

**Note:** *CT-SCZ* clozapine-treated schizophrenia patients, *RT-SCZ* risperidone-treated schizophrenia patients, *NT-SCZ* never-treated schizophrenia patients, *HCs* healthy controls, *NS* not significant, *, Significant group difference at *P* < 0.05; **, *P* < 0.01; ***, *P* < 0.001

### Relationships with clinical symptoms

The volumes of the mid-anterior CC region and the FA values of the anterior CC region in patients were not significantly associated with PANSS total or subscale scores.

## Discussion

To the best of our knowledge, this is the first study to date investigating the impact of long-term monotherapy of clozapine and risperidone on callosal structure in chronic schizophrenia. Our analyses demonstrated significant reductions of callosal volumes in NT-SCZ in comparison with HCs, which were located in the central and mid-anterior region of the CC. We also found that FA values of the anterior CC region in NT-SCZ significantly reduced compared to HCs. Moreover, both RT-SCZ and CT-SCZ showed significantly increased FA values in the anterior CC region compared to NT-SCZ, and only RT-SCZ had larger volume in the mid-anterior CC region compared with NT-SCZ. Together, these findings suggest that chronic exposure to clozapine and risperidone may have different effects on callosal structure in schizophrenia.

The findings of our present study agree well with other studies in the literature demonstrating that chronic schizophrenia patients without any antipsychotic medications were associated with smaller volumes and FA values of the CC in comparison with HCs [3, 17, 19–21]. As an extension of prior findings, one important observation here is that callosal deficits were mainly located in the anterior and mid-anterior CC region. White matter fibers passing through these subregions are responsible for connecting the frontal lobes [22, 23]. Therefore, anatomical alterations in the anterior and mid-anterior region of the CC may be related to structural and functional abnormalities of the frontal lobe in schizophrenia, which generate delusions and hallucinations [24, 25]. However, other studies showed that chronic schizophrenia patients had more widespread deficits that were located in both anterior and posterior region of the CC [3, 19, 20]. This discrepancy may be due to sample heterogeneities related to race, handedness, sex, and age, and to methodological differences [3]. Apart from anterior and mid-anterior CC, we also observed significant volume reduction in central CC in never-treated patients compared with HCs, which is in line with findings from Collinson et al. [17]. Furthermore, it is suggested that alterations of this subregion with time in schizophrenia may be associated with stage of illness [17]. Thus, it is interesting to explore whether structural alterations in central CC are a stable biomarker to predict the developmental trajectory of schizophrenia in future studies.

The observation that micro- and macrostructural abnormalities of the CC in schizophrenia were located in different subregions is in line with other two studies [19, 24]. Here, we found that alterations of callosal volume were in the mid-anterior and central region, and abnormalities of white matter integrity were only in the anterior region of the CC. It has been proposed that changes in the number of axons and the degree of myelination may lead to the changes in white matter volume but not anisotropy [17, 26, 27]. Since the index of anisotropy is related with fiber integrity [28, 29], its change in schizophrenia patients may relate to disruptions of oligodendrocytes and/or myelin surrounding axons because of inflammation or dysregulations of neuroinflammatory responses [30–33]. Our findings suggest different pathophysiological processes in these subregions of the CC.

The current findings, together with findings from previous studies, suggest that chronic exposure to antipsychotics may have an impact on anatomical organization of the CC in schizophrenia [11, 12]. In this study, both CT-SCZ and RT-SCZ showed significantly increased FA values in the anterior CC region in comparison with NT-SCZ. Prior studies have demonstrated that antipsychotic medications may modulate inflammations and immune responses through reduced activation of microglia and macrophages, increased level of anti-inflammatory cytokines, and inhibition of the release of proinflammatory cytokines [34, 35]. And animal studies in the past decade showed that chronic exposure to antipsychotic medications attenuated white matter lesions [36–39]. Therefore, clozapine and risperidone may repair microstructural deficits of the CC by reducing inflammation or immune responses. Interestingly, only schizophrenia patients receiving long-term risperidone, not clozapine, showed significantly increased volumes of mid-anterior CC. Evidences from gene expression profiling and neuroimaging studies support the hypothesis that dysregulation of the dopaminergic system is associated with myelination impairment in psychiatric disorders [40, 41]. It has been suggested that risperidone and clozapine have different affinity for dopamine receptors [10]. Bartzokis et al. also found that antipsychotic medications differently mitigated myelination deficits in schizophrenia depending on their affinities to dopamine receptors [8, 42, 43]. Therefore, our findings may suggest that different impacts of two drugs on callosal volumes are related to their different abilities in modulating the dopaminergic system.

There are several limitations in the present study. First, because that this study is a cross-sectional design and it lack of random assignments to different antipsychotic medications for schizophrenia patients, it should be careful to interpret our current findings. However, it is unethical and challenging to request the patients to receive a single antipsychotic treatment for over five years in a longitudinal study with a randomize design. Therefore, our cross-sectional design may be a feasible method to explore the effects of long-term usage of antipsychotics on human brain. Second, the sample size in this study is relatively small, and may be not completely representative. Third, the usage of non-antipsychotic medications was not controlled according to individual clinical condition. Four, as the most widely used index of DTI, FA essentially combines information about diffusion direction and diffusion speed thus provides an overall summary of fiber integrity [44]. Therefore, FA is our primary parameter of interest. In addition, more diffusion parameters will be explored in the future studies.

## Conclusions

Using comparing schizophrenia patients with long-term mono-antipsychotic medications with un-medicated chronic patients and HCs, the present study displays a more severe change of callosal anatomy in never-treated patients and demonstrates differential alterations of callosal structure in patients with different antipsychotic treatments. These findings suggest that the pathology itself is responsible for cerebral abnormalities in schizophrenia and that chronic exposure to antipsychotic medications may have an impact on white matter structure of schizophrenia patients, especially in those with risperidone treatment.

## Data Availability

The datasets generated and analysed during the current study are not publicly available due to their containing information that could compromise patients’ privacy, but are available from the corresponding author on reasonable request.

## Abbreviations

ANOVA: analysis of variance
ANCOVA: analysis of covariance
CC: corpus callosum
CT-SCZ: clozapine-treated schizophrenia patients
DTI: diffusion tensor imaging
FA: fractional anisotropy
FDR: false discovery rate
HCs: healthy controls
MANCOVA: multivariate analysis of covariance
MRI: magnetic resonance imaging
NT-SCZ: never-treated schizophrenia patients
PANSS: Positive and Negative Symptom Scale
RT-SCZ: risperidone-treated schizophrenia patients
TIV: total intracranial volume.
